# The Role of Oxidative Stress in Etiopathogenesis of Chemotherapy Induced Cognitive Impairment (CICI)-“Chemobrain”

**DOI:** 10.14336/AD.2015.1022

**Published:** 2016-05-27

**Authors:** Amelia Maria Gaman, Adriana Uzoni, Aurel Popa-Wagner, Anghel Andrei, Eugen-Bogdan Petcu

**Affiliations:** ^1^Research Center of Experimental and Clinical Medicine, University of Medicine and Pharmacy of Craiova, 200349, Romania; ^2^Filantropia City Hospital Craiova, Romania; ^3^Department of Psychiatry, University of Medicine Rostock, 18147 Rostock, Germany; ^4^Biochemistry Department, University of Medicine and Pharmacy “Victor Babes” Timisoara; ^5^Griffith University School of Medicine, Gold Coast Campus, Griffith University, QLD 4222, Australia

**Keywords:** cancer, chemobrain, chemotherapy induced cognitive impairment, aging, oxidative stress

## Abstract

Chemobrain or chemotherapy induced cognitive impairment (CICI) represents a new clinical syndrome characterised by memory, learning and motor function impairment. As numerous patients with cancer are long-term survivors, CICI represent a significant factor which may interfere with their quality of life. However, this entity CICI must be distinguished from other cognitive syndromes and addressed accordingly. At the present time, experimental and clinical research suggests that CICI could be induced by numerous factors including *oxidative stress*. This type of CNS injury has been previously described in cancer patients treated with common anti-neoplastic drugs such as doxorubicine, carmustine, methotrexate and cyclophosphamide. It seems that all these pharmacological factors promote neuronal death through a final common pathway represented by TNF alpha (tumour necrosis factor). However, as cancer in general is diagnosed more commonly in the aging population, the elderly oncological patient must be treated with great care since aging *per se* is also impacted by oxidative stress and potentiually by TNF alpha deleterious action on brain parenchyma. In this context, some patients may develop cognitive dysfunction well before the appearance of CICI. In addition, chemotherapy may worsen their cognitive function. Therefore, at the present time, there is an acute need for development of effective therapeutic methods to prevent CICI as well as new methods of early CICI diagnosis.

*Chemobrain or chemotherapy induced cognitive impairment (CICI)* represents a newly described clinical diagnosis associated with cancer therapy. This neuropsychological syndrome induced by pharmacological agents used in oncological therapy is centred on the gradual cognitive decline of the patients which could range from mild inability in performing some tasks to serious attention and memory problems [1-3]. In fact, CICI should be regarded as a pharmacological side-effect characterised by deficits in memory function and concentration which are seen at least 5-10 years after cessation of chemotherapy [4-8].

The combination of electroencephalography (EEG) with multimodal neuroimaging (anatomical MRI and fMRI assessments) have added to the understanding of the underlying mechanisms of chemotherapy-induced cognitive impairment. Neuroimaging may help uncover a neural basis for the subtle cognitive deficits in cancer patients. However, a serious limitation of functional imaging studies in CICI is the consistent use of small samples [9].

A significant number of experimental and clinical studies have reported neuropsychological symptoms after chemotherapy with platinum compounds, mitotic spindle and proteasome inhibitors as well as interferon alpha and small thyrosine kinase inhibitors [10,11]. More specifically, CICI was described in several studies focusing on patients with breast cancer treated with doxorubicin and cyclophosphamide. These patients presented with impairment in memory acquisition and learning as well as impairment of their visual-spatial skills. Overall all the breast cancer patients that had chemotherapy, presented with low cognitive scores requiring significantly more time to perform a task as compared with the pre-chemo stage [4,8,12-14]. However, only a limited number of studies have evaluated the impact of this newly described pathological entity on the quality of life. Selamat MH *et al*. (2014) have evaluated cognitive deficits in breast cancer patients after chemotherapy on quality of life and reported that medical practitioners fail to diagnose and address appropriately this condition as well as the whole array of associated features experienced by the chemotherapy patients including but not limited to calls for help and coping, daily life and survivorship issues [15]. Moreover, a recent clinical case cohort study conducted in breast cancer patients treated with cyclophosphamide, methotrexate and 5-fluorouracil revealed that cognitive deficits induced by these agents can last for more than 20 years [16-18]. Therefore, CICI needs a better clinical identification and management considering that a significant number of cancer patients are long-term survivors. This is also upheld by the fact that some of the most important features described in these patients relate to motor CNS functions and memory. A meta-evaluation of 30 studies including more than 838 chemotherapy patients and controls reported significant changes in verbal memory and executive motor functions which can interfere significantly with the quality of life of these patients [19]. We assume that CICI could potentially interfere with their compliance, ultimately affecting their medical management.

The above mentioned abnormal neuropsychological changes correlate with morphological changes recorded by neuro-structural radiological tests. A study investigating structural changes in brain parenchyma in breast chemotherapy patients has reported a time dependent gradual continuous structural damage. In addition, a significant reduction in total brain and grey matter volumes was recorded post-chemotherapy in breast cancer survivors [16,17,20]. These findings suggest that neuropsychological changes could be determined by direct CNS tissue deterioration induced by various pharmacological factors used in cancer therapy.

Functional magnetic resonance imaging (fMRI) studies investigating changes associated with cerebral blood flow dependent blood oxygenation level (BOLD) have shown that patients with breast cancer have a different CNS activation pattern as compared with healthy controls during declarative memory tasks. More specifically, after therapy with cyclophosphamide, methotrexate, and 5-fluorouracil, their pre-frontal cortex presents with a lower degree of activation during memory formation. The authors report a significantly lower activation in the dorso-lateral and caudal frontal cortex as well as in the pre-motor cortex. The anomalies recorded in the left caudal lateral frontal cortex were positively linked to a superior executive dysfunction but unexpectedly, during recall, cancer patients have a greater level of generalised diffuse activation compared with healthy subjects. [21,22]. These results have been reinforced by the studies of Baudina et al. [23], who have reported significant changes in glucose metabolism in several brain areas after chemotherapy. The changes were more obvious in the frontal lobes, where the authors recorded a metabolic impairment which was proportional to the amount of pharmacological agents [23]. Presently it is suggested that numerous other pharmacological and non-pharmacological factors are capable to promote psychological changes indicative of cognitive dysfunction. Therefore, CICI should be correctly diagnosed and managed.

The mechanisms for chemotherapy induced cognitive changes are unknown, but several mechanisms have been proposed, including the direct neurotoxicity of chemotherapeutic agents, genetic predisposition (APOE4, COMT), oxidative damage, chemotherapy induced peripheral neuropathy, immune dysregulation or shortened telomeres [24-27] (Fig. 1). However, some authors suggest that at least in colon cancer patients treated with FOLFOX4, there is no CICI. The mild cognitive changes described in these patients could be explained by the psychological adaptive changes related to the actual cancer diagnosis as well as the associated anxiety [28]. These findings have been recently contradicted by a study that evaluated verbal memory in colon cancer treated with FOLFOX4. This study reports a significant acute decrease in verbal memory in more than 50% of patients. At least 33% of all patients developed a subsequent worsening of their cognitive function [29]. Therefore, it is safe to admit that at least theoretically any type of chemotherapy could promote CICI.

## Cognitive dysfunction in cancer patients

Cognitive impairment described in cancer patients is determined by numerous factors. Perhaps the most important is related to the origin and location of the tumour. We can assume that a patient with a primary or metastatic lesion in the CNS could present with neuropsychological changes at an early stage compared with a patient presenting with a tumour located in another organ/system and without brain secondary deposits. Other factors contributing to cognitive dysfunction in cancer are represented by associated diseases especially those requiring anti-depressive and pharmacological therapy for pain. Also, the aging factor must be carefully considered as cancer is encountered in significant numbers in an aging population. In this setting, preliminary changes suggestive of dementia could be already present before cancer development and chemotherapy. Therefore, there is an acute need to separate the cognitive dysfunction induced by chemotherapy (CICI), from those neuropsychological changes induced by aging, co-morbidities and associated therapies.

**Figure 1.**
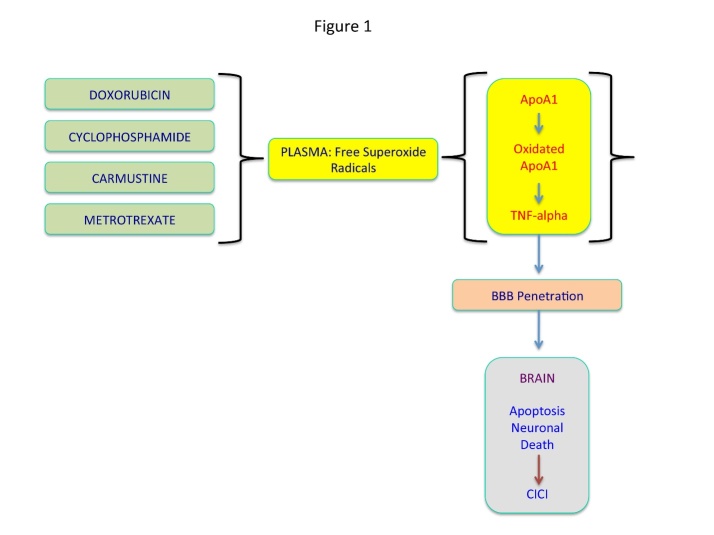
Mechanisms underlying CICI Chemotherapy of cancer generates free superoxide radicals in plasma which may cause the oxidation of Apolipoprotein A1 (ApoA1). Oxidated ApoA1 in turn promotes synthesis of TNF alpha which penetrates the blood brain barrier to further inflict apoptosis and neural death in the brain.

In the realm of *chemotherapy induced cancer impairment (CICI)*, the oxidative stress represents one of the most common etio-pathological factor leading to significant neurobiological changes. More specifically, the cognitive dysfunction in this setting may be directly promoted by the reactive oxygen species (ROS) generated during chemotherapy. Butterfield DA (2014) [30] reports that doxorubicin, one of the most common anti-neoplastic agents, generates superoxide free radicals in plasma which then oxidate apolipoprotein A1 (ApoA1). ApoA1 in turn promotes synthesis of TNF alpha which acts on its receptors located on blood brain-barries (p55 and p75) and subsequently reaches the brain parenchyma where it inflicts apoptosis and neural death (Fig. 1) [31]. However, it is of paramount importance to note that a significant number of chemotherapeutic drugs exert their anti-neoplastic action by inducing oxidative stress in malignant tissue as well as in the brain and other organs and systems. In brain parenchyma, morphological changes suggestive of oxidative stress injury after cyclophosphamide, one of the most common chemotherapeutic agents, are represented by significant abnormalities in granular and neuronal dendrites as well as spine density and immaturity of spines [32].

## Deleterious effects associated with oxidative stress

Activated oxygen species, especially those that share electronic orbital features with singlet oxygen, are highly reactive, and therefore, the evolutionary adaptation by organisms to make oxygen wholly beneficial has never been complete. Consequently, there are many deleterious effects associated with excessive, activated ROS [33-35]. Production of cellular reactive oxygen species (ROS) is typically associated with protein and DNA damage, toxicity, and neuronal death and are intimately linked to mutated proteins with mitochondria [36]. These effects are more evident when ROS are in excessive concentrations and the ability of antioxidant mechanisms of neurons to counterbalance the damaging reactions is diminished [37].

The brain is especially susceptible to the assaults perpetrated by ROS. This is because the brain is a big oxygen consumer (20% of the body consumption), causing it to be especially vulnerable to oxidative stress due to its low level of protective antioxidant mechanisms. The brain contains a large amount of polyunsaturated peroxidizable fatty acids along with high levels of iron that act as pro-oxidant and sometimes induces autophagic cell death. In addition, lipid peroxidation leads to the production of toxic compounds such aldehydes or dienals (e.g. 4-hydroxynonenal) which may cause in turn, neuronal apoptosis [38].

At DNA level, oxidative modifications may cause rapid depletion of intracellular energy by activating DNA repair enzymes. Energy scarcity after stroke will cause in turn, endonuclease-mediated DNA fragmentation, the key mechanism that leads to DNA damage [39].

## Chemotherapy induced oxidative stress in brain

It was postulated that CICI represents the ultimate result of synergistic action of several factors including chemotherapy mediated oxidative stress, direct vascular and neurotoxic action of some chemotherapeutic agents as well as inefficient DNA-repair mechanism(s) [25,26].

Oxidative stress derived ROS may be the most important factor promoting CICI and it seems that some patients may have a genetic predisposition to develop this condition. A study conducted in a children diagnosed with acute lymphoblastic leukaemia suggests that cognitive dysfunction after oxidative stress induced by chemotherapy is associated with polymorphisms in three oxidative stress genes, NOS3 894T, SLCO2A1 variant G allele and GSTP1 variant allele [40]. The role of the oxidative stress in initiating and maintaining brain damage has been suggested by previous studies which have reported that a normal redox state is a very important factor modulating the functionality of CNS including differentiation of oligodendrocyte-type-2 astrocyte progenitor cells [41]. In CICI, chemotherapeutic agents are capable to induce a state of oxidative stress via an increased synthesis of plasma cytokines capable to reach the CNS after passing through the blood brain barrier [12]. It is generally accepted that the most important cytokine released during chemotherapy in plasma is TNF alpha which may promote direct oxidative injury to the CNS leading to neuronal damage [42]. The most important chemotherapeutic drugs inducing *through TNF alpha* a state of oxidative stress and subsequent CICI are presented below.

*Doxorubicin.* Doxorubicin, an anthracycline anti-tumour antibiotic indicated in several types of malignancies, is known to generate intracellular oxygen species as its effects on heart are explained by the ROS synthesis [12, 43]. In vitro studies have demonstrated that neurons treated with this drug will show evidence of protein and lipid oxidation. Moreover, experimental studies conducted in a murine model have shown that doxorubicin promotes a significant level of generalised CNS oxidative stress which is substantiated by increased levels of protein oxidation as well as lipid peroxidation in brain parenchyma [29]. Subsequent research has proved that doxorubicin has an indirect CNS toxic action as a result of an increase in plasma TNF alpha which penetrates though blood-brain barrier. It is suggested that plasmatic TNF alpha is produced in significant amounts as a result of oxidation of APOA1 by doxorubicin which in turn stimulates TNF alpha production by macrophages [44].

In the brain, TNF alpha produces a significant oxidative stress associated with a reduction in glutathione levels, a known anti-oxidant, in parallel with increased levels of glutathione peroxidase and reductase as well as increased glutathione-S-transferase levels. In addition, redox proteomics evaluation suggested a significant CNS oxidative stress induced via TNF alpha production by doxorubicin [45]. Additional studies revealed that plasma doxorubicin- mediated TNF alpha promotes a significant decrease in brain mitochondrial respiration as a result of an increase in p53, Bax and associated apoptosis [46]. All of these suggest that TNF alpha represents a crucial therapeutic target in patients with CICI.

Experimental work conducted in a murine model has revealed that simultaneous administration of the anti-oxidant 2-mercaptoethane sulfonate (MESNA) prevents oxidative damage induced by doxorubicin. More importantly, it seems that MESNA is capable to inhibit the increase in peripheral TNF alpha induced by doxorubicin [44]. A recent clinical study conducted in patients with breast cancer and non-Hodgkin’s lymphoma has demonstrated that MESNA simultaneously administered with doxorubicin reduces the levels of TNF alpha, TNF-receptor 1 and TNF-receptor 2. Interestingly, it seems that those patients with the highest level of TNF alpha before chemotherapy would benefit the most of MESNA therapy [47]. Unfortunately, the authors have not evaluated cognitive changes in this cohort of patients. Therefore, further research is needed to evaluate any neuropsychological changes which could be explained by MESNA administration.

*Carmustine*. Studies conducted in rats have revealed that carmustine induces a major oxidative stress level in the brain parenchyma as well as an impairment of memory and learning processes. Administration of carmustine is associated with a significant increase in plasma TNF alpha and CNS malondialdehyde, a marker indicating significant oxidative stress. Histological evaluation has also shown an over-expressed caspase-3. Remarkably, administration of carmustine in a rat model induces an increased synthesis of metallothionein in hippocampus [48]. This is rather unexpected as this factor has an anti-oxidative protective role. The significance of an increased induction of metallothionein by carmustine is yet to be thoroughly investigated.

Ultimately, it seems that oxidative stress produced by reactive oxygen species (ROS) after carmustine administration, are produced as a result of inhibition of glutathione reductase. Also, ROS species activate c-jun N-terminal kinase (JNK) and extracellular signal-regulated kinase (ERK) pathways which finally will promote neurotoxicity [49].

*Methotrexate.* Methotrexate is a dihydrofolate reductase inhibitor used in the treatment of cancer, including lymphomas and breast cancer. Methotrexate is capable to produce nephrotoxicity subsequent to reactive oxygen species (ROS) formation. The role of oxidative stress in this setting is suggested by an increased level of plasma TNF alpha [50]. Research conducted on a murine model has demonstrated that a standard dose of methotrexate has a significant effect on spatial and non-spatial memory tests. These abnormal cognitive tests could be explained by the functional changes induced by methotrexate in frontal lobes and hippocampus [51]. These studies were followed by clinical reports that highlighted the fact that in children with acute lymphoblastic leukaemia, methotrexate induces oxidative stress in CNS membrane phospholipids and CNS tissue injury which could explain perfusion deficits, atrophy and cognitive neuro-psychological cognitive changes [52]. Remarkably, the oxidative stress markers induced by methotrexate such as oxidated phosphatidylcholine may be recovered in cerebral spinal fluid of patients with cognitive dysfunction [53].

More information related to the full extent of oxidative stress induced by methotrexate have been revealed by other experiments. In an animal model, it induces lipid peroxidation in the plasma as well as a significant increase in HSP70 and glutathione reduction in several brain regions [54]. Research conducted in a murine model injected with a breast cancer cell line (FM3A) revealed cognitive dysfunction and depression after methotrexate administration. Also, methotrexate significantly increased the levels of several pro-inflammatory factors such as COX2 and iNOS. Interestingly, it decreased the population of progenitor cells in hippocampus which could explain the cognitive impairment noted in these subjects inoculated with breast cancer cells [55].

*Cyclophosphamide*. This anti-cancer factor is recommended in various combinations in a wide variety of malignancies. In a standard dosage, it may induce neurotoxicity but administered in a high dosage it may cause confusion and visual blurring [56]. Rarely, in cases of non-Hodgkin lymphoma, the cyclophosphamide-containing CHOP regimen may induce a reversible posterior leukoencephalopathy syndrome or a fatal necrotizing leukoencephalopathy syndrome [57-59].

Experimental research has shown that cyclophosphamide administered intra-peritoneally induces a significant oxidative stress in the CNS. This is quantified by an increased level of brain malondialdehyde, a product of lipid peroxidation and oxidation of fatty polyunsaturated acids [60]. It seems that the deleterious oxidative action of cyclophosphamide is achieved via increased release of TNF alpha and IL6. In parallel, after administration of cyclophosphamide there is an increased production of COX2, iNOS, NfkB and p38-MAPK [61]. In addition, cyclophosphamide inhibits activity of brain, heart and lung catalase as well as the superoxide dismutase and anti-oxidant potential of plasma. Also, cyclophosphamide decreases the gluthatione levels [62].

Interestingly, administration of Annatto seeds (Bixa orella), linseed oil (Linum usitatissimum) and boron to animal models can counter act the oxidative stress effect induced by cyclophosphamide. However, this has not yet been verified in a clinical setting in patients treated with cyclophosphamide [60,62,63]. Remarkably, a recent study conducted in a murine model of cyclophosphamide-induced CICI has reported that the intra-hippocampal transplantation of human stem cells reverse all neuropsychological changes associated with cyclophosphamide administration [64]. This results may be extrapolated to other pathological settings characterised by cognitive impairment but more exploratory and validation studies are needed before designing a pilot study.

## The elderly patient with CICI: a cautionary note

Cancer is a multi-factorial disease acquired as a result of numerous acquired genetic and molecular abnormalities. As more and more people are living longer they are prone to develop these molecular hits which will further result in the development of malignant lesions. However, with the advent of new therapeutic options including chemotherapy, elderly patients diagnosed with cancer are nowadays long-term survivors. As a result of chemotherapy, we can assume that these subjects will sustain a generalised/CNS oxidative stress injury which will induce a more or less severe chemotherapy induced cognitive impairment (CICI)/chemobrain. This needs adequate assessment and therapy as it could interfere significantly with the quality of life of elderly cancer patients including their medical compliance.

However, if CICI supervenes on a background of systemic aging, the brain aging factor is of paramount importance as it may accentuate the overall cognitive impairment. Remarkably, both CNS aging and CICI are induced and accelerated by oxidative stress and ROS. Therefore, a thorough understanding of molecular pathways and factors modulating oxidative stress is of paramount importance as it may lead to discovery and synthesis of factors specifically designed to improve cognition, motor function and quality of life in elderly patients with CICI.

## CNS aging

Fifty years ago, Brody H (1955) [65] has reiterated the idea that normal brain aging or senescence is characterised by a cognitive decline and a significant brain weight reduction subsequent to wide spread neuronal death in several cerebral areas including the frontal lobe and hippocampus [65]. These have been confirmed by several modern neuro-radiological studies but the actual molecular and genetic background related to neuronal death is entirely understood [66]. However, Ge et al (2002) [67] suggest that normal brain aging is associated with reduction in both white and grey matter. However, while the grey matter is reduced linearly in parallel with advancing age while white matter decreases quadratically. The most significant reduction is recorded only after middle-age. Therefore, it might be plausible that white matter is more sensitive to oxidative stress induced by aging and determines the inter-individual cognitive variability in patients with CICI [67]. Interestingly, a recent neuroimaging review suggest that in patients with CICI there is a decrease in both white and grey matter volumes as well as a decrease in frontal lobe activity [68]. This confirms the neuronal death theory of Brody [65]] related to frontal lobe changes. However, these data would suggest that both CICI and CNS aging could start initially induced the same cerebral region by oxidative stress.

In aging, neurons and glial cells increase their ability to cope with stress, producing new neurons and glial cells and remodelling neuronal circuits. Increased ROS levels and accumulation of oxidation products derived from nucleic acids, proteins and lipids, may induce mitochondrial dysfunction, perturbation of different metabolic and signalling pathways, cause endothelial damage of brain micro-vessels. Subsequently, these abnormalities are followed by progressive changes in hemodynamic stability predisposing to inflammatory and ischemic changes [69,70]. Overall there is a massive reduction of cerebral vasculature and angiogenesis potential of the brain which contributes to brain atrophy [70,71]. Sonntag et al. (1997) [72] suggests that the reduction of the cerebral vascular network associated with aging is determined by a significant drop in growth hormone and insulin-like growth factor 1 [72].

**Figure 2.**
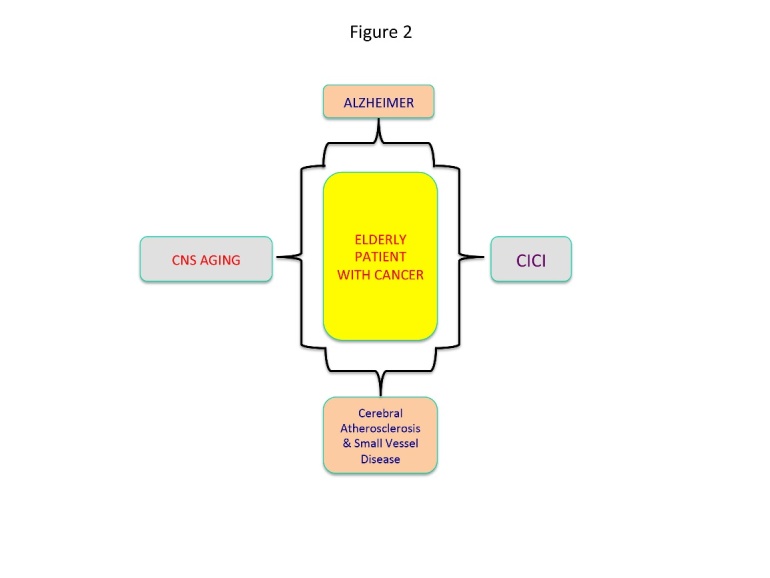
Pathological conditions developed in elderly Oxidative stress in old age leads to several pathological entities characterized by cognitive dysfunction.

Moreover, prior to CICI, elderly patients could develop several pathological conditions associated with cognitive decline which are more commonly described in their age group. This population may develop Alzheimer’s disease as a result of complex mutations or any other vascular cerebral condition. The most important vascular entities associated with cognitive impairment are the cerebral atherosclerosis and small vessels disease. In both settings oxidative stress and ROS are the most important pathogenic factors facilitating injury to the vessel wall by accumulation of oxidation products. The natural biology of these pathological conditions is characterised by brain tissue haemorrhages and infarcts which would increase the cognitive impairment. Also Alzheimer’s disease is associated with amyloid angiopathy which could also lead to brain ischemia and subsequent neuronal death, ultimately amplifying cognitive impairment [73,74] (Fig. 2).

## Oxidative stress and Aging

Aging or normal senescence is a physiological, multi factorial process modulated epigenetically and characterised by the accumulation of neurotoxic molecules in cells and tissues. It is also characterised by mutations in repair genes and telomere shortening [75]. During aging, multiple molecular, cellular, structural and functional changes occur in all organs including the brain which is characterised a decline of its integrity and impairment of memory and motor function control. The final insult in brain senescence is represented by the neuronal dysfunction and death. In this setting, oxidative stress, mitochondrial dysfunction, oxidative cell damage to DNA, proteins and lipids, inflammatory process, altered cell signalling pathways, apoptosis and changes in gene expression are important factors contributing to the aging process [76].

The Harman’s hypothesis of free radicals claims that free radicals cause progressive accumulation of oxidative damage to lipids, proteins as well as DNA, promoting cellular damage induced by ROS. More specifically, the ROS attack glial cells and neurons, leading to neuronal damage and apoptosis [77]. According to Harman’s theory, the interplay between ROS and protective antioxidant defence systems represents an important factor in modulating the process of aging and ultimately the life-span [78-83]. Complementary to the free radical theory of aging, the theory of mitochondrial free radical theory suggests that aging is determined by accumulation of clonal errors related to mtDNA replication. In this context, mitochondria plays a fundamental role amplifying the oxidative stress that drives the aging process [84]. Many studies have shown that 8-oxo-dG (7,8-dihydro-8-oxo-2′-deoxyguanosine (8-oxo-dG), a product resulting from DNA oxidation, is seen in higher levels associated with mtDNA than nuclear DNA. This would indicate that mtDNA is more susceptible to oxidative damage than nuclear DNA [85-90].

Both mitochondrial dysfunction and mitochondrial metabolism modulate the process of aging, through several molecular pathways represented by the insulin/IGF-1 signalling (IIS), the target of rapamycin (mTOR) and the respiratory chain. Abnormalities in these molecular pathways will led to an overproduction of ROS [70]. The mitochondrial free radical theory of aging relies on the fact that mitochondrial function and the activity of several ROS- scavenging enzymes decline with age while the ROS production increases. However, oxidative damage affects the replication and transcription of mitochondrial DNA (mtDNA), increasing the prevalence of mutations/deletions and partakes in a vicious cycle as the somatic mtDNA mutations impair the respiratory chain function. Finally, the increased ROS production promotes more oxidative damage to mtDNA, proteins and lipids [83,84]. Remarkable, animal studies have shown that sleep fragmentation associated with old age promotes the transfer of TNF-alpha across blood brain barrier [91]. Although, these data have to be verified in clinical studies, they are of paramount importance as it would suggest that aging promotes brain alterations via TNF alpha final effector pathway, similar with some chemotherapeutic factors (Fig. 2).

## Conclusions

Oxidative stress is one putative mechanism underlying chemotherapy-induced cognitive impairment associated with cancer treatment. As a significant number of those receiving chemotherapy are long term survivors this issue could significantly interfere with their quality of life. A special attention should be dedicated to the elderly patient with cancer undergoing chemotherapy. The aging process is associated with morphological and functional changes in the brain which may be also modulated by oxidative stress. Oxidative stress or changes in redox state are implicated in age-related malfunction of the brain. An increase in oxidative stress or decreases in the antioxidant capacity of the brain are key factors involved in neuronal degeneration in the elderly [37]. In addition, the elderly represent a population group at increased risk for cognitive deficits. Considering that at least in the Western world, the average life-span is continuously increasing, we expect that the number of elderly patients with CICI will increase significantly leading to serious socio-economic implications. Finally, we note that the "chemobrain effect" may also be transitory and very mild, as reported for drugs such as Oxaliplatin and Fluorouracil [28] but nevertheless there is an acute need for effective pharmacological agents and protocols to prevent CICI as well as new methods of early diagnosis which at the present time are lacking. Moreover, it is important to highlight that the evidence provided by preclinical models, in vivo or in vitro, should be evaluated with caution. The therapy evaluated in these models may not mirror the schedules usually administred to oncological patients. It is worth remembering that the total accumulated dose can be similar in patients and animal models but the pharmacological effect may not be the same.
